# Suction Cups‐Inspired Adhesive Patch with Tailorable Patterns for Versatile Wound Healing

**DOI:** 10.1002/advs.202100201

**Published:** 2021-07-01

**Authors:** Rongkang Huang, Xiaoxuan Zhang, Wenzhao Li, Luoran Shang, Hui Wang, Yuanjin Zhao

**Affiliations:** ^1^ Department of Colorectal Surgery and Provincial Key Laboratory of Colorectal and Pelvic Floor Diseases Guangdong Institute of Gastroenterology Sixth Affiliated Hospital of Sun Yat‐sen University Guangdong 510655 China; ^2^ Department of Rheumatology and Immunology The Affiliated Drum Tower Hospital of Nanjing University Medical School Nanjing 210008 China; ^3^ State Key Laboratory of Bioelectronics School of Biological Science and Medical Engineering Southeast University Nanjing 210096 China; ^4^ Department of Biomedical Engineering The Hong Kong Polytechnic University Hung Hom Kowloon Hong Kong 999077 China; ^5^ Zhongshan‐Xuhui Hospital The Shanghai Key Laboratory of Medical Epigenetics Institutes of Biomedical Sciences Fudan University Shanghai 200032 China

**Keywords:** bioinspired patches, biomaterials, selective adhesion, tailorable patterns, wound healing

## Abstract

Medical patches play an important role in wound healing because of their tissue conformality, drug release capacity, and convenient operation. Great efforts have been devoted to developing new‐generation patches with distinctive features promoting wound healing. Here, inspired by the structure of octopus suction cups and the component of natural tissue, a biocompatible wound patch with selective adhesiveness and individualized design using a combined strategy of template‐replication and mask‐guided lithography is presented. Such patches are based on Ecoflex film with suction‐cup‐mimicking microstructures to adhere to normal skin and with biocompatible gelatin methacryloyl (GelMA) hydrogel to contact wounded areas. An ultraviolet mask with a tailorable pattern is employed to shape the GelMA hydrogel into customized geometry replicating individual wound areas, and thus both adhesion and antiadhesion properties are integrated into the same patch. In addition, vascular endothelial growth factor is loaded to accelerate the healing process. Based on these advantages, the authors demonstrate that the present patches not only adhere to different skin surfaces, but also promote the treatment of a rat cutaneous wound model. Thus, it is believed that this versatile patch can break through the limitation of traditional patches and be ideal candidates for wound healing and related biomedical applications.

## Graphical abstract

A new class of selective adhesiveness, tailorable patterns, and individualized design wound patch is prepared by synergizing the suction cup‐inspired Ecoflex films and good biocompatible gelatin methacryloyl hydrogels using a combined strategy of template‐replication and mask‐guided lithography.

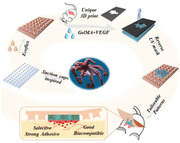

## 1 Introduction

Wound healing has attracted remarkable attention in recent years for their severe challenges and serious economic burdens.^[^ ^1^ ^]^ To realize this goal, a variety of strategies have been developed, such as debridement, drainage, glycemic control, wound patches, and so on.^[^ ^2^ ^]^ Among them, patches that can adhere to the wounded skin and release drugs are considered as one of the ideal stratagems of accelerating wound healing.^[^ ^3^ ^]^ The necessary properties of patches usually include good biocompatibility, effective adhesion, and flexible applicability.^[^ ^4^ ^]^ Although many successes have been achieved in this area, rare patches could achieve these properties at the same time. Generally, most existing adhesive patches are made of bioharmful chemical derivative components and may induce allergic response, secondary skin damage, and contamination,^[^ ^5^ ^]^ while patches from biosafe polymers typically have poor mechanical match with tissues.^[^ ^6^ ^]^ In addition, most of the current synthesis methods only generate wound patches with uniform shapes, bringing difficulties in exactly covering a unique wound site of each individual.^[^ ^7^ ^]^ Therefore, multifunctional wound patches with strong adhesive and good biocompatibility for precision treatment is still anticipated for wound healing.

**Figure 1:**
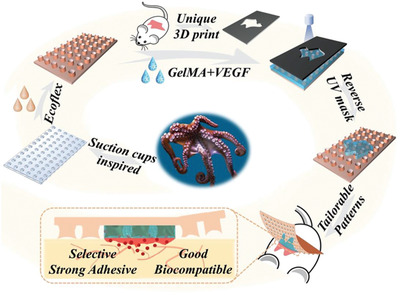
Schematic illustration of the bioinspired skin‐adhesive patch with tailorable wound dressing. Unique GelMA–VEGF dressing on the surface of the Ecoflex patch was formed with the integration of a UV mask with an individual wound shape.

In this paper, inspired by the structure of octopus suction cups and the component of natural tissue,^[^ ^8^ ^]^ we present novel composite gelatin patches with excellent biocompatibility, superior adhesive ability, and tailored patterns for versatile wound healing, as schemed in **Figure** **1**. The cup‐shaped protruding chambers of octopi, also called suction cups, could adapt to and conformably seal different surfaces through physical adhesion forces.^[^ ^8b^ ^]^ Specifically, the dome‐like protuberances of the suction cups provide extremely low internal pressure relative to the ambient pressure, which results in strong and reversible adhesion performances against both dry and wet surfaces.^[^ ^9^ ^]^ Benefitting from these attributes, various artificial adhesives with miniaturized suction cups have been designed and processed for various applications in clean transfer systems, soft robotics, wearable devices, stimuli‐responsive adhesives, and so on.^[^ ^10^ ^]^ However, the application of these suction‐cup‐inspired systems in developing skin adhesive patches for wound healing is seldomly explored.

Herein, we developed a suction‐cup‐mimicking patch with selective adhesiveness, individualized graph design, stretch ability, and good biocompatibility through template‐replication and mask lithography. The patches were based on a silicone rubber (Ecoflex) film with suction‐cup microstructures to adhere to normal skin and with biocompatible gelatin methacryloyl (GelMA) hydrogel to contact wounded areas. Attributed to the suction‐cup microstructures on the Ecoflex film, the patches could exhibit strong adhesion toward both dry and wet surfaces; while GelMA, which has remarkable similarity to the extracellular matrix, endowed the patches with excellent biocompatibility.^[^ ^11^ ^]^ Besides, vascular endothelial growth factor (VEGF) was added in the GelMA hydrogel to further accelerate the healing process.^[^ ^12^ ^]^ Particularly, as a ultraviolet (UV) mask with a tailorable pattern was employed during the fabrication process, the GelMA hydrogel was shaped according to the random geometry of individual wound areas, and thus the opposite properties of adhesion and antiadhesion were integrated into a single patch film. Based on these features, it was demonstrated that such patches could precisely cover individual wound areas and effectively promote wound healing via a rat cutaneous wound model. These results indicated that this versatile composite patch could be ideal candidates for achieving precision treatment of wound healing in clinical medicine.

## 2 Result and Discussion

**Figure 2:**
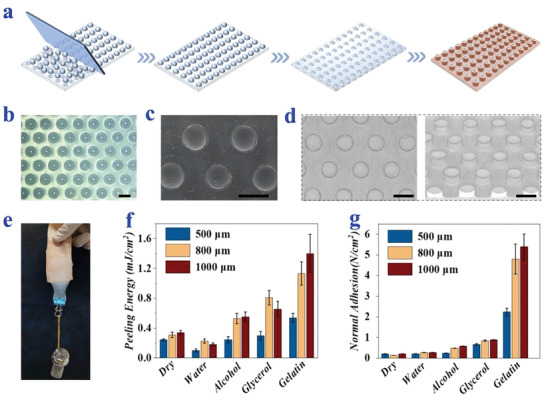
Fabrication and measurement of the adhesion ability of the bioinspired patches. a) Schematic diagram of the fabrication process of the bioinspired patch. b,c) Optical b) and SEM c) images of the suction cups (800 µm). d) Micro‐CT images of the suction cups (800 µm) from top view and front view. The scale bars are all 1000 µm. e) A photograph showing of the adhesive patch attached to pigskin fully supporting a 0.2 kg weight. f,g) The peeling‐off f) and pull‐off g) strengths of three different diameters of suction cups against dry and wet surfaces.

In a typical experiment, suction cup‐inspired patches were fabricated by replicating a specially designed negative mold. The negative mold was composed of an ordered array of microcolumn cavities (Figure S1a, Supporting Information). Specifically, a gravity‐based self‐assembly method was adopted to make monodispersed steel microspheres sink into the microcolumns. A wiper blade was used to ensure that every single microsphere was filled and fixed. The radius of the microspheres was slightly larger than that of the cavities to obtain the negative mold with a cambered bottom (**Figure** **2**a; and Figure S1b,c, Supporting Information). Ecoflex, a silicone rubber with good biocompatibility and elasticity in tension compression (Figure S2, Supporting Information) was used for manufacturing the patch by replicating the structure of the negative mold. Premixed and uncured Ecoflex liquid precursor was poured to the surface of the mold, which fully filled the microcolumns with cambered bottoms. After vacuum degassing, thermal curing, and demolding, the micro suction‐cup array was finally obtained (Figure 2b–d). It was worth noting that Ecoflex patches with different sizes could be fabricated simply by changing the dimension of the negative mold, indicating the tailorability of this method (Figure S1a and S3, Supporting Information).

We then investigated the adhesion ability of the as‐prepared patch toward both dry and wet surfaces. Several studies have proven that wetting the interface can effectively enhance the adhesion of the suction cups, especially on rough surfaces.^[^ ^8^, ^9^ ^]^ Generally speaking, when a liquid diffuses at an interface and forms a uniform film, the contact area of the interface is maximized compared to the solid–solid interface, resulting in the absorption of the source of capillary force. In addition, when a negative pressure is formed in the cavity, the liquid molecules at the interface will prevent external fluids from entering and play a role in maintaining the negative pressure. These effects would be even more obvious in rough interfaces, such as pigskin. Here, we selected four clinically‐used liquids (water, ethanol, glycerin, and gelatin) to wet the interface. The results showed that Ecoflex patch achieved a prominent adhesion performance with wet surfaces, as they could withstand a total weight of 0.2 kg tangentially when vertically adhering to a wet pigskin (Figure 2e). The data of normal adhesion and peeling energy also proved the above hypothesis (Figure 2f,g). For practical applications in constructing versatile skin‐adhesives, the patch's adhesion performances in pull‐off and peeling directions were measured against dry and different wet surfaces. It was found that the patches with different suction cup diameters (500, 800, and 1000 µm) all showed stronger adhesion on wet surfaces compared to those against dry surfaces (Figure 2f,g). In addition, the pull‐off adhesion forces in both circumstances showed that the array of the microsuckers with 800 or 1000 µm size had similar adhesion and both were higher than that of 500 µm (Figure S4a, Supporting Information). For a simple column array and flat patch, only the effect of capillary force contributes to adhesion, and thus the adhesion capacity was smaller (Figure S4b,c, Supporting Information).

**Figure 3:**
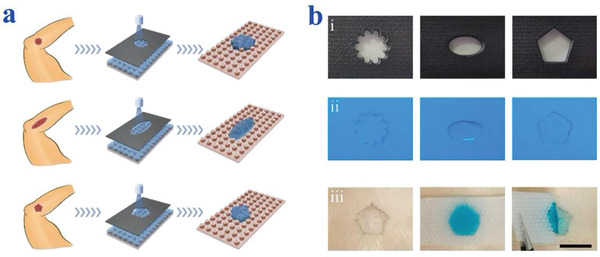
Fabrication of GelMA dressing with tailorable shapes. a) Schemes of the fabrication of GelMA dressing with tailorable shapes. b,i) Different shapes of UV masks generated by 3D printing. ii) Fabrication of GelMA hydrogels with tailorable shapes according to the UV masks. iii) The GelMA hydrogel could precisely cover individual pigskin wound areas, and the opposite properties of adhesion and antiadhesion are integrated into the same patch film. Blue dye was added into the GelMA solution for better imaging. The scale bar is 1000 µm.

In addition to adhesion properties, biocompatibility is another important concern for the biomedical application of a patch. To promote wound healing, GelMA was chosen as a good biocompatible dressing, as the composition and structure of GelMA hydrogel well resemble the natural extracellular matrix. GelMA has a low fracture energy and normal adhesion (37 kPa) and is sensitive to cracks (Figure S2, Supporting Information). The Fourier Transform Infrared Spectroscopy (FTIR) spectrum of GelMA hydrogel was shown in Figure S5 (Supporting Information), where gelatin showed its characteristic peak of C─H stretching at 2935 cm^−1^ and C═O stretching at 1632 cm^−1^. In addition, porous network structure of the GelMA hydrogel was characterized through scanning electron microscopy (SEM) (Figure S6, Supporting Information). With the Ecoflex patches as the substrate, individual shape of GelMA hydrogel could be prepared facilely through mask lithography, as schemed in **Figure** **3**a. Specifically, a UV mask was first designed by 3D printing according to the random shape of individual wound, which could block part of the UV light while allowing the other part to pass through. Then, GelMA solution was filled on the surface of the Ecoflex patch and was covered with the mask. Therefore, when irradiated by UV light, only pregel solution at specific regions exposed to UV would polymerize to form a solid hydrogel, whereas the unexposed part still kept in liquid form and could be swept away. Thus, tailorable hydrogel dressings were achieved according to different shapes of UV masks (Figure 3b). In addition, different concentrations of GelMA hydrogels were prepared and their performances in constructing unique shape were assessed. Results showed that a series of GelMA hydrogel with different pregel concentrations (10, 15, and 20 wt%) could form unique shape easily and consistently (Figure S7, Supporting Information).

**Figure 4:**
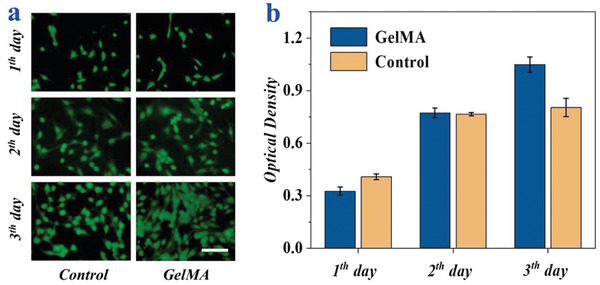
Biocompatibility assay of GelMA. a) The confocal laser scanning images of 3T3 cells cultured on glass or GelMA hydrogels after 3 days, the scale bar is 50 µm. b) The results of the MTT assay of 3T3 cells cultured on different substrates for 3 days.

The biocompatibility of GelMA was investigated by a standard 3‐(4,5‐dimethylthiazol‐2‐yl)‐2,5‐diphenyltetrazolium bromide (MTT) assay. Specifically, 3T3 cells cultured with the GelMA hydrogel was set as the experimental group, and the cells cultured in blank wells was set as the control group. It was verified that the cells could be distributed on the surface of the blank wells homogenously after 24 h of culture (**Figure** **4**a). The MTT assay results were consistent with the cell fluorescent images (Figure 4b). The cells in all groups could remain the same favorable metabolic activity over 3 days. In addition, the good biocompatibility of the GelMA hydrogels was verified by the increased cell viability with the culturing time, which demonstrated that these hydrogels could provide an extracellular matrix (ECM)‐mimicking environment and stimulate host cell adhesion and proliferation.

To investigate the drug release profile of the hydrogel, fluorescein isothiocyanate labeled bovine serum albumin (FITC–BSA) was utilized as a model to simulate the release of VEGF.^[^ ^4c^ ^]^ GelMA hydrogel has been reported to degrade in several days in vivo.^[^ ^13^ ^]^ Thus, with the degradation of GelMA, preload VEGF could be released from the GelMA hydrogel and promote wound healing. Therefore, with the degradation of the GelMA hydrogel, both VEGF and BSA would be released in the solution at a comparable rate due to their similar molecular weight (VEGF 40 kDa, BSA 66.5 kDa) and hydrodynamic radius.^[^ ^14^ ^]^ Specifically, GelMA hydrogel containing FITC–BSA was exposed to different concentrations of collagenase II solution, and the drug release profile was determined by measuring the optical density (OD) value of the solution. It was found that the release of FITC–BSA could reach a peak value quickly within 10 h (Figure S8, Supporting Information). This indicated that the GelMA hydrogel could release active molecules at the early stage of the recovery of skin wound.

**Figure 5:**
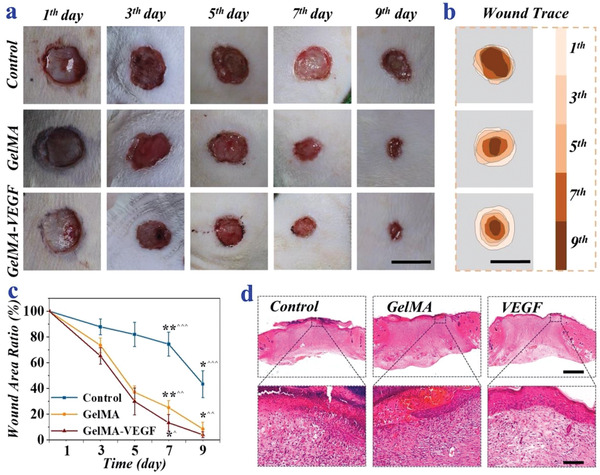
Rat wound healing model and H&E staining. a) Images of the wounds treated with PBS solution (control), GelMA, and GelMA–VEGF. b) The wound trace of three groups. c) The wound area ratio of three groups, * *P* < 0.05, ** *P* < 0.01 (one‐way ANOVA followed by Bonferroni's multiple comparison test), “^”: GelMA–VEGF group versus GelMA group, “^^”: control group versus GelMA group, “^^^”: control group versus GelMA–VEGF group. d) Representative H&E staining of the wounds after 9 days. Scale bars are 1.0 cm in a) and b), 1000 µm (first row) and 100 µm (second row) in d), respectively.

To demonstrate the practical value of the GelMA hydrogel, the in vivo wound healing assay was performed by creating 1.0 cm round wounds on the back of rats. The rats were then treated with GelMA hydrogel, GelMA‐VEGF hydrogel, and PBS solution (control group), respectively. For the in vivo wound healing experiments, since the group treated with the GelMA/VEGF shows almost complete wound closure at day 9, so the change of the wound areas was recorded and characterized at day 0, 3, 5, 7, and 9. It was found that the wound areas in all three groups were gradually reduced (**Figure** **5**a). For visualization, the dynamic healing process was traced and illustrated in the schematic diagram in Figure 5b. These results showed that the wounds treated with GelMA hydrogel could achieve significant healing rate than the control group (Figure 5c). The wound area ratio was significantly lower in the GelMA group than the control group at day 7 (25.1% vs 74.4%, *P* < 0.01) and day 9 (8.5% vs 43.4%, *P* < 0.05), respectively. This could be attributed to good biocompatibility and cell adhesion ability of the GelMA hydrogel. More prominently, the GelMA‐VEGF group had an even lower wound area ratio at day 7 (12.6% vs 74.4%, *P* < 0.01) and day 9 (4.0% vs 43.4%, *P* < 0.05) compared to the control group. It thus demonstrated the benefit of VEGF by comparing the wound area ratio of the GelMA‐VEGF group and the GelMA group after treatment for 7 days (12.6% vs 25.1%, *P* < 0.05). The microscopic examination of hematoxylin‐eosin (H&E) staining was further conducted to investigate the constriction of the wound beds as well as the progress of epithelization. On day 9, the control group showed more inflammatory cells infiltration, more local hemorrhagic focus, and looser connective tissue than the GelMA group and the GelMA‐VEGF group. In addition, the results demonstrated that the regenerated epithelial tissues were thicker and more intact in the GelMA‐VEGF group than the other two groups (Figure 5d). These results also supported that the GelMA‐VEGF group had much better wound healing effect than the GelMA group and the control group.

To investigate the biological mechanism of the wound healing process, Masson's trichrome staining and immunohistochemistry staining were carried out, respectively.

**Figure 6:**
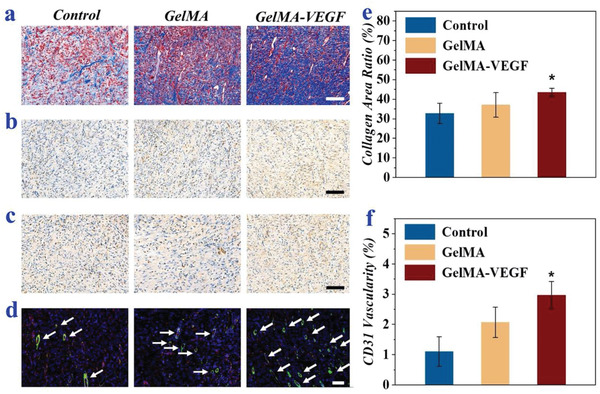
Investigation of the biological mechanism of the wound healing process. a) Masson staining images of the wounds of different groups (control, GelMA, and GelMA–VEGF) on day 9. b,c) Corresponding immunohistochemistry staining of IL‐6 b) and TNF‐*α* c) on day 9. d) Immunofluorescence of CD31/*α*‐SMA on day 9. CD31^+^ structures (red) were surrounded by *α*‐SMA positive cells (green) in different groups. The arrows indicate the vascular ducts. e,f) Quantitative analysis of the collagen density e) and blood vessel density f) on day 9, * *P* < 0.05 versus control group. The scale bars are all 100 µm.

The growth and deposition of collagen are important for wound healing. Masson's staining showed that the collagen deposition was denser and more organized in both two groups containing GelMA (**Figure** **6**a). Apparently, the GelMA patch with VEGF achieved significantly greater deposition of collagen than the control group (Figure 6e). Besides, the immunohistochemistry staining of interleukin (IL)‐6 and tumor necrosis factor (TNF)‐*α* showed that fewer infiltration of IL‐6 and TNF‐*α* was observed in the GelMA‐VEGF group (Figure 6b,c). In addition, the quantitative analysis results demonstrated that the GelMA‐VEGF group achieved a significant lower infiltration of IL‐6 compared with that of the control group (*P* < 0.05, Figure S9, Supporting Information). Except for the collagen deposition, angiogenesis is another necessary index during the remolding of tissue. Thus, double immunofluorescence staining of CD31 and alpha smooth muscle actin (*α*‐SMA) were performed to indicate neovascularization. It was worth noting that the density of vascular structure was obviously different in the three groups (Figure 6d). For the control group, few positive results of CD31 and *α*‐SMA immunostaining was observed, while the other two groups presented a higher expression level of CD31 and *α*‐SMA owing to the presence of GelMA. In addition, GelMA‐VEGF group possessed a significant higher density of neo‐vessels compared with those of the control group (*P* < 0.05, Figure 6f). The accelerated angiogenesis was probably due to the early release of VEGF from GelMA, which stimulated proliferation and migration of endothelial cells.

Although GelMA hydrogel show cell adhesion, GelMA has a low fracture energy and normal adhesion (37 kPa) and is sensitive to cracks (Figure S2, Supporting Information), which is much lower than soft tissues such as skin (tensile modulus is 2–140 MPa).^[^ ^15^ ^]^ Soft tissues are not sensitive to cracks due to the staggered distribution of internal collagen fibers. What's more, after contacting with the wound surface, GelMA hydrogel gradually swells and degrades (Figure S8, Supporting Information), and its mechanical properties further decline. Therefore, when the patch was pulled off from the wound surface, the GelMA hydrogel would break before inducing secondary damage to the tissue. All the results of faster wound closure, tissue regeneration, angiogenesis, and safety demonstrated that the GelMA hydrogel, together with the VEGF, showed satisfying therapeutic effects. Therefore, the tailorable bioinspired patch shows a high potential in promoting the healing of skin wound, which could be an ideal precision treatment strategy for tissue regeneration.

## 3 Conclusion

In summary, inspired by octopi, we have developed a biocompatible and tailorable would patch for individualized wound healing. Such bioinspired patches were fabricated by combining the template replication method with the mask‐guided lithography technology. The patches were constituted by Ecoflex films integrated with GelMA hydrogels. The Ecoflex films had suction‐cup‐like microstructures and exhibited a superior adhesion capability which was demonstrated by in vitro adhesion test. The GelMA hydrogel possessed good biocompatibility and could promote cell adhesion and proliferation when loading with VEGF, which was confirmed by in vivo wound healing assay. Besides, customized UV masks were utilized to form tailorable patches. These features indicate that the bioinspired and tailorable wound patch may provide insights in developing skin‐attachable medical devices for precision treatment in wound healing and other biomedical fields.

## 4 Experimental Section

### Materials, Cell Lines, and Animals

Gelatin (from porcine skin), methacrylic anhydride (94%), and dimethyl sulfoxide were purchased from Sigma‐Aldrich, USA. GelMA hydrogel was made in the laboratory from gelatin and methacrylic anhydride. VEGF was purchased from Pepro Tech, USA. MTT reagent and collagenase‐II were provided by Thermo Fisher Scientific, USA. Phosphate buffered saline (1x PBS) solution was prepared from a 10x solution purchased from Beijing Solarbio Science & Technology Co., Ltd., China. Silicone rubber liquids (Ecoflex) were obtained from Smooth‐On, Inc., USA. Steel microspheres were purchased from Changzhou Linwang Driving Medium Co., Ltd., China. Stainless steel plates with microcolumns were purchased from Dongguan Yuantong Screen Co., Ltd., China. High Glucose Dulbecco's Modified Eagle's Medium, fetal bovine serum, penicillin–streptomycin double antibiotics and trypsin‐ethylene diamine tetraacetic acid (EDTA) were all obtained from Gibco, USA. FITC‐BSA was purchased from Solarbio, Shanghai, China. A Millipore Milli‐Q system provided deionized water with a resistivity of 18 MΩ cm^−1^ for the whole experiment.

Standard fibroblast cell line (NIH 3T3) cells were supplied by the Cell Bank of the Chinese Academy of Sciences, Shanghai, China. The Sprague‐Dawley rats (200–250 g in weight) were provided by Hangzhou Ziyuan Laboratory Animal Technology Co., Ltd., China.

### Characterization

Digital images of the hydrogel, patch, pork skin, UV mask, and Sprague‐Dawley rats were captured by a digital camera (Canon, EOS 700D, Japan). The SEM and Micro‐CT images were characterized using a field scanning electron microscope (FESEM, UltraPlus, Zeiss) and microcomputed tomography (SkyScan 1176, Bruker), respectively.

### Production of UV Masks

First, the random shape of an individual wound, such as oval, pentagon, and flower‐shape was recognized and drawn by an imaging process software and stored in “.stl” format. UV masks were then 3D printed with nylon material by WeNext Technology Company, China.

### Fabrication of Ecoflex Patch

The two precursor liquids of Ecoflex were mixed uniformly with a mass ratio of 1:1 according to the instructions and poured on the surface of the negative mold which fully filled the microcolumns with cambered bottoms, forming ≈1.0 mm thick liquid surface. The template was put into a vacuum drying oven to degas until no bubbles were generated. This process lasted about 1 min. After that, it was put into an oven for thermal curing at 80 °C within 2 h and 100 °C within 1 h. After the template was taken out and cooled to room temperature, the patch was carefully demolded, the micro suction‐cup array was finally obtained.

### Mechanics Performance Testing—Material Tensile Test

The Ecoflex or the GelMA hydrogel were made into a film with a thickness of 1.0 mm and cut into a dumbbell shape, respectively. The length of the dumbbell's neck was 10.0 mm, and the width was 5.0 mm. It was then loaded on an electronic tensile testing machine (HP‐500, Handpi) and stretched at a speed of 1.0 mm min^−1^ until it broke to get the final stress–strain curve and tensile modulus.

### Mechanics Performance Testing—Material Compression Test

Ecoflex was made into a cylinder with a diameter and height of 1.3 cm. It was loaded on an electronic tensile testing machine (HP‐500, Handpi) and compressed at a speed of 1.0 mm min^−1^ until it reached 50% strain (the compression displacement at this time was 0.65 cm, and the height of the deformed material was 0.65 cm). The final stress–strain curve and compressive modulus were then obtained. After removing the pressure, the height of the material was measured. Recovery rate was ratio of height before and after compression.

GelMA was made into a cylinder with a diameter and height of 1.3 cm. It was loaded on an electronic tensile testing machine (HP‐500, Handpi) and compressed at a speed of 1.0 mm min^−1^ until it is broken. The final stress–strain curve and compressive modulus were then obtained.

### Mechanics Performance Testing—Peeling‐Off Test

The adhered surface (glass slide or pigskin) was fixed on the lower work fixture of an electronic tensile testing machine (HP‐500, Handpi). Pork skin bought from the local butcher's was first cut into 4.0 × 2.0 cm^2^ pieces. The required medium was spread evenly on the interface to infiltrate. Different kinds of Ecoflex patches with the area of 3.5 × 1.5 cm^2^ were placed on the surface of the pork skin pieces and then were pressed with the force of 5.0 N for 15 s. After that, the patches were lifted off with the initial peeling angle of about 10° and the displacement speed at 0.1 mm s^−1^. The total peeling off energy during this process was the integral of the force‐displacement curve which was recorded and calculated by a bundled software (MulForce). The peeling off energy per unit area was the value divided by the total area of the patch.

### Mechanics Performance Testing—Normal Adhesion Test

Similar to Peeling off, the patch of 3.5 × 1.5 cm^2^ and the adhered surface (glass slide and pigskin) were fixed on the upper and lower work fixture of an electronic tensile testing machine (HP‐500, Handpi), respectively. The required medium was spread evenly on the interface to infiltrate. Then a pressure of 5.0 N was preapplied and maintained for 15 s, and measuring was started with the displacement speed at 0.1 mm s^−1^ after the zero adjustment to obtain a force‐displacement curve. Normal adhesion was the ratio of the maximum suction force to the patch area in the process.

### Preparation of GelMA Solution

GelMA (10, 15, 20 wt%) was dissolved in deionized water at 40 °C and mixed with 1.0 wt% photoinitiator to obtain the pregel solution. 0.25 µg mL^−1^ VEGF was added to the GelMA pregel solution and exposed to UV light for 5.0 s to obtain the GelMA–VEGF hydrogel.

### Fabrication of Tailorable GelMA Hydrogel Dressing

Three shapes of black UV masks (oval, pentagon, and polygon) were prepared by 3D printing. Different concentrations of GelMA solution (10, 15, 20 wt%) were prepared and filled on the plastic dish. Blue dye was added into the GelMA solution for better imaging. After exposure to UV light covered with UV mask within 5.0 s, only pregel solution at specific regions would become hydrogel, whereas the unirradiated part still kept in liquid form and was swept away by PBS. Thus, different shapes of GelMA hydrogels were achieved.

### In Vitro Drug Release Experiment

To investigate the release activity of VEGF in GelMA hydrogel, 0.5 mg mL^−1^ FITC–BSA was mixed with the GelMA solution to fabricate FITC–BSA loaded GelMA hydrogel (GelMA/FITC–BSA). Round GelMA/FITC‐BSA hydrogels with a diameter of 20.0 mm and a thickness of 1.0 mm were tested for drug release, and put into 10.0 mL PBS solution with different concentration of collagenase‐II (1.0, 0.5, 0.25 mg mL^−1^), which were placed in an incubator equipped with a rotary shaker and incubated for over 54 h (200 rpm, 37 °C). After a predetermined time (0, 1, 2, 3, 4, 6, 8, 10, 12, 18, 24, and 30 h), 1.0 mL of the medium was taken out from the release buffer and replaced by 1.0 mL of fresh PBS solution. The amount of FITC–BSA released from GelMA hydrogel was evaluated by a microplate reader (Thermo Fisher Scientific, USA), and the drug release profile was drawn accordingly.

### In Vitro Cell Experiments

To conduct the MTT assay, NIH 3T3 cells were divided into two groups and cocultured with two different materials in a 24‐well plate for 3 days, respectively. The first group was only glass slides; the second group was pure GelMA films of the same size. Each group for each day had triple parallels, and the initial concentration of cells in each well was 4 × 10^5^ cells mL^−1^. The assay was performed according to the manufacturer's recommendations. Cell viability for each group was acquired by measuring the corresponding OD values by a microplate reader (Thermo Fisher Scientific, USA). Cell‐laden hydrogels with live/dead staining were analyzed with a fluorescent microscope (Olympus, IX50, Olympus Corporation, Tokyo, Japan).

### In Vivo Animal Experiment

All rats were treated following the Laboratory Animal Care and Use Guidelines strictly. All the experimental operations involved in animals were reviewed and approved by the Animal Ethics Committee of South China Agricultural University. Eighteen Sprague‐Dawley rats were randomly and equivalently divided into three groups. Their backs were shaved, and a rounded full‐thickness cutaneous wound area with the diameter of 1.0 cm was created. The first group was the control group, in which the rats received no treatments except PBS solution. The second was the GelMA group and the third was the GelMA–VEGF group. The recovery of wounds was recorded on days 0, 3, 5, 7, and 9. For visualization, the dynamic healing process was traced and drawn by ImageJ and PowerPoint softwares. On day 9, all rats were sacrificed, and tissues over the wound bed were removed and immersed in neutral formaldehyde. Histological analysis was tested by Wuhan Servicebio Technology Co., Ltd, China.

### Statistical Analysis

All statistical analysis were conducted using SPSS software. Data were expressed as mean ± standard deviation. Statistical differences were determined using one‐way analysis of variance (ANOVA). The levels of significance were labeled with *p < 0.05, **p < 0.01.

## Conflict of Interest

The authors declare no conflict of interest.

## Author Contributions

Y.J.Z. conceived the idea and designed the experiment; R.K.H. and W.Z.L. conducted experiments and data analysis; R.K.H., W.Z.L., X.X.Z., L.R.S., and Y.J.Z. wrote the manuscript. H.W. assisted with the scientific discussion of the article.

## Supporting information

Supporting Information

## Data Availability

The data that supports the findings of this study are available in the supplementary material of this article.
