# Supplementary material for: Suction Cups‐Inspired Adhesive Patch with Tailorable Patterns for Versatile Wound Healing
Source: Adv Sci (Weinh). 2021 Jul 1;8(17):2100201. doi: 10.1002/advs.202100201 (PMC8425934; doi:10.1002/advs.202100201)
Supplement: Supplementary file 1 — Supporting Information [file ADVS-8-2100201-s001.pdf]

© 2021 Wiley-VCH GmbH

ADVANCED SCIENCE
Open Access

# Supporting Information

for *Adv. Sci.,* DOI: 10.1002/advs.202100201

# Suction cups-inspired Adhesive Patch with Tailorable Patterns for Versatile Wound Healing

*Rongkang Huang, Xiaoxuan Zhang, Wenzhao Li, Luoran Shang*, Hui Wang*, Yuanjin Zhao**

# Supplementary

**Suction cups-inspired Adhesive Patch with Tailorable Patterns for Versatile Wound Healing**

Rongkang Huang[a], Xiaoxuan Zhang[b,c], Wenzhao Li[d], Luoran Shang[e,*], Hui Wang[a,*], Yuanjin Zhao[b,c,*]

[a] Department of Colorectal Surgery and Provincial Key Laboratory of Colorectal and Pelvic Floor Diseases, Guangdong Institute of Gastroenterology, Sixth Affiliated Hospital of Sun Yat-sen University, Guangdong 510655, China

[b] Department of Rheumatology and Immunology, The Affiliated Drum Tower Hospital of Nanjing University Medical School, Nanjing 210008, China

[c] State Key Laboratory of Bioelectronics, School of Biological Science and Medical Engineering, Southeast University, Nanjing 210096, China

[d] Department of Biomedical Engineering, The Hong Kong Polytechnic University, Hung Hom, Kowloon, Hong Kong, China

[e] Zhongshan-Xuhui Hospital, the Shanghai Key Laboratory of Medical Epigenetics, Institutes of Biomedical Sciences, Fudan University, Shanghai 200032, China

Email: luoranshang@fudan.edu.cn; wang89@mail.sysu.edu.cn; yjzhao@seu.edu.cn

**Simulation calculation of single suction-cup geometry**

When microspheres are filled into microcolumns and become tangent, the

cross-sectional view is as follows (Figure S1). At this time, the radius of the microsphere is $r_s$, the radius of the microcolumn is $r_c$, and the height is $h_1$. The height from the bottom of the suction cup to the bottom of the arc is $h_2$, and the central angle is $\boldsymbol{\theta}$. The center angle at this time is $\theta = 2\arccos(r_c/r_s)$ . $h_2 = h_1 + \sqrt{r_s^2 - r_c^2} - r_s$ .

Mark the number of microspheres per square centimeter as n and the area of micro suction-cup as S ($cm^2$). The mass of each microsphere is m (g). The microspheres of mass $M = n * s * m$ (g) are weighed and dumped evenly over the area of S ($cm^2$), which is pre-enclosed. The system is then slightly disturbed by tools such as sweeping by the tablet. The gravitational potential energy difference before and after each microsphere filling each microcolumn is equal to $-\Delta E_p = mg(h_1 - h_2)$ , and each microsphere will partially fill the microcolumn and be tangent to it as it tends to the lowest energy. The self-assembly process is thus completed.

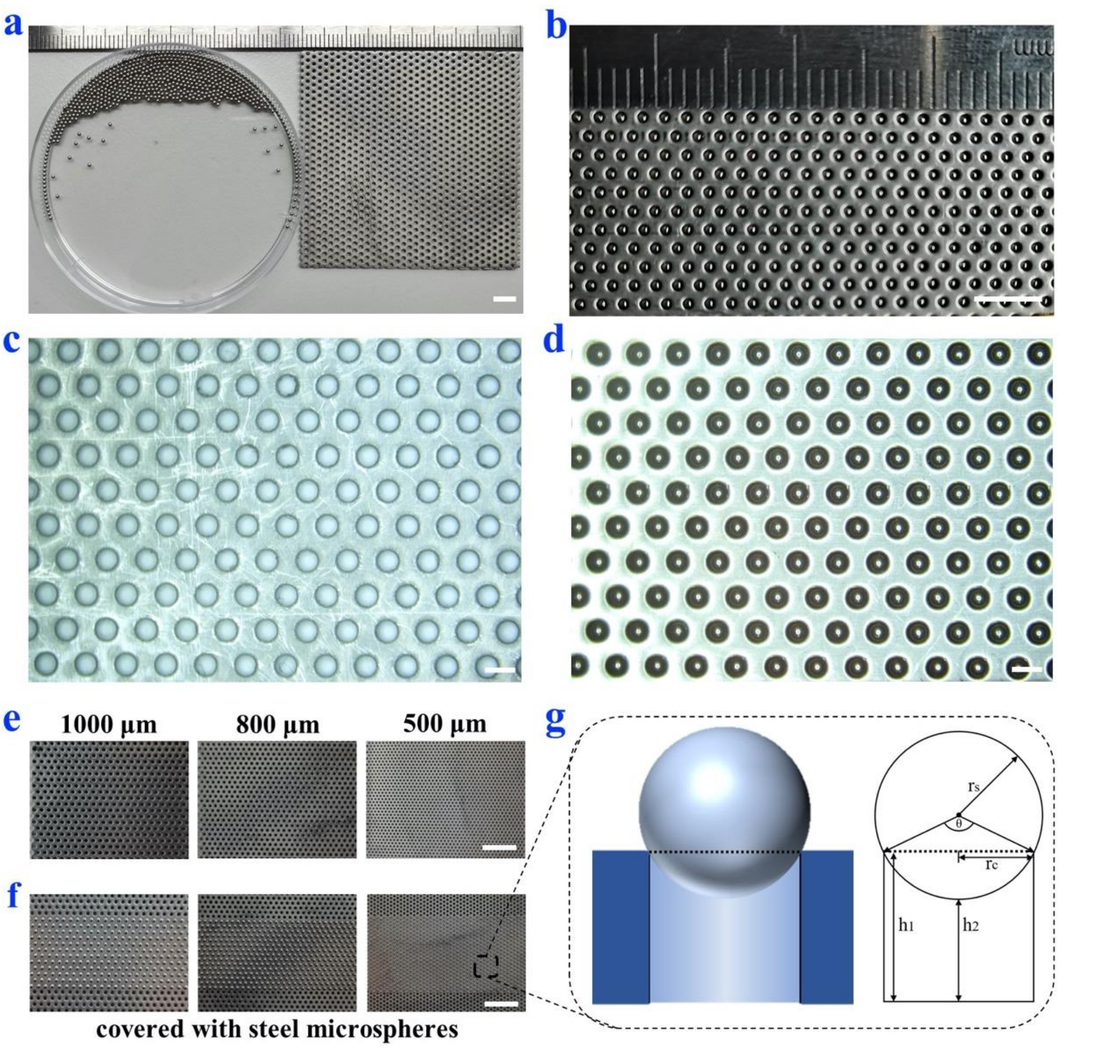


**Figure S1.** Generation of the negative mold. (a,b) Digital photograph of steel microspheres and the empty negative mold (a), as well as the negative mold covered with steel microspheres (b). The scale bars are 0.5 cm. (c,d) Optical microscopic images of the empty negative mold (c) and the negative mold covered with steel microspheres (d). The scale bars are all 1.0 cm. (e) Different sizes of negative molds (1000 μm, 800 μm, and 500 μm). (f) Different sizes of microspheres (1100 μm, 900 μm, and 600 μm) were employed to occupy the bottom cavity of the microcolumns of the negative mold with the help of a wiper blade. The scale bars are all 1.0 cm. (g) Schematic diagram of the suction cup geometry.

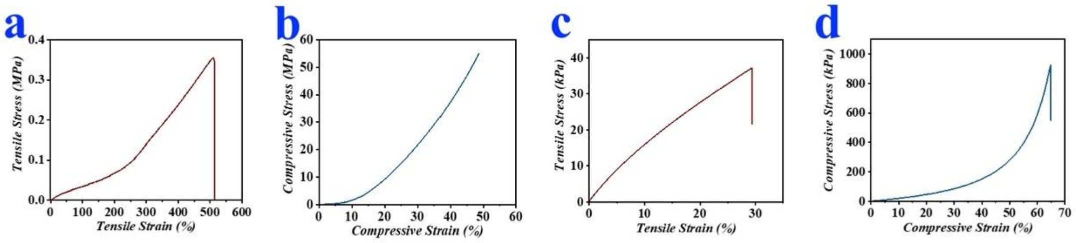


**Figure S2.** (a) Tensile stress-strain curves of the Ecoflex patch. (b) Compressive stress-strain curves of the Ecoflex cylinder. (c) Tensile stress-strain curves of the GelMA hydrogel. (d) Compressive stress-strain curves of the GelMA hydrogel.

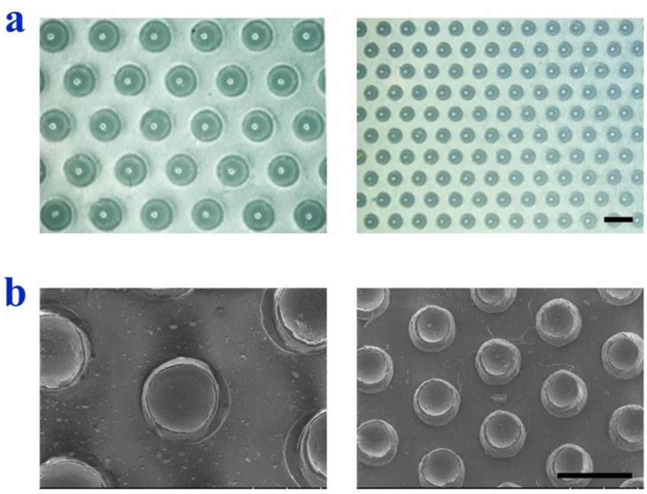


**Figure S3.** (a) Optical microscopic images of the suction cups (1000 μm and 500 μm). (b) SEM images of the suction cups (1000 μm and 500 μm). The scale bars are all 1.0 cm.

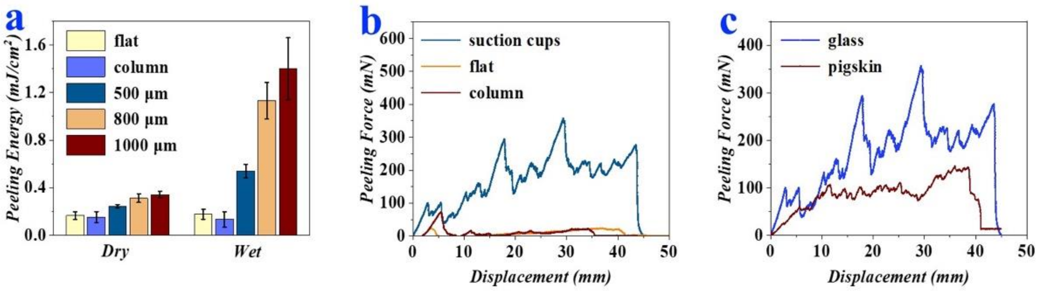


**Figure S4.** (a) Dry and wet adhesion strengths for Ecoflex patches with various arrays

(flat, column, 500 μm, 800 μm, and 1000 μm). (b) Wet surface (gelatin) adhesion strengths with various arrays (suction cups, flat, and column). (c) Adhesion strengths of suction cups (800 μm) for glass and pigskins.

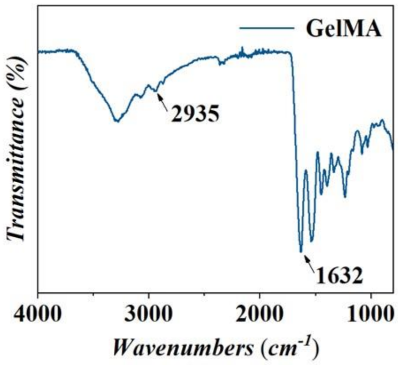


**Figure S5.** FTIR of GelMA. Gelatin shows its characteristic peak of C-H stretching at 2935 $cm^{-1}$ and C=O stretching at 1632 $cm^{-1}$.

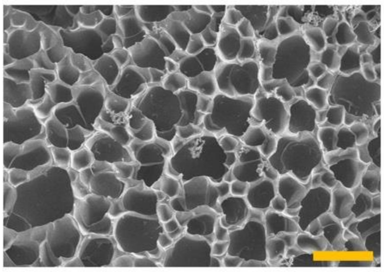

**Figure S6.** SEM images of the cross section of the GelMA hydrogel, the scale bar is 20 μm.

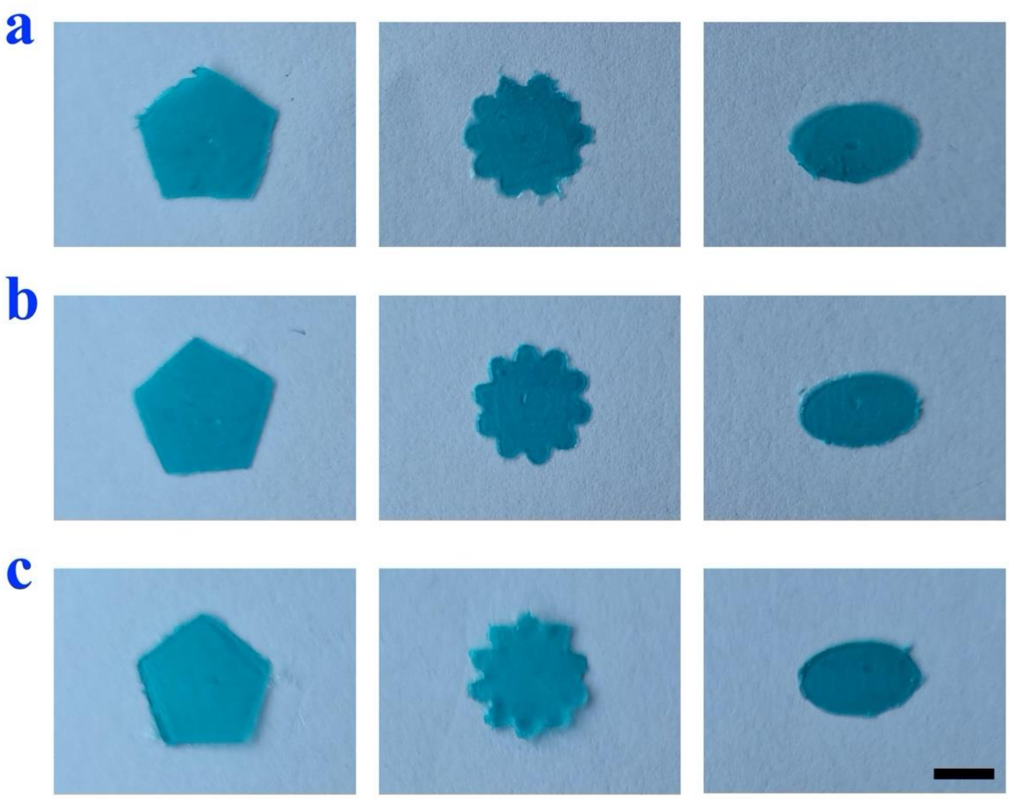


**Figure S7.** Different concentrations of GelMA hydrogels with tailorable patterns. (a-c) GelMA hydrogels fabricated by mask-guided lithography technique with concentrations of 10 wt% (a), 15 wt% (b), and 20 wt% (c), respectively. Blue dye was added into the GelMA solution for better imaging. The scale bar is all 0.5 cm.

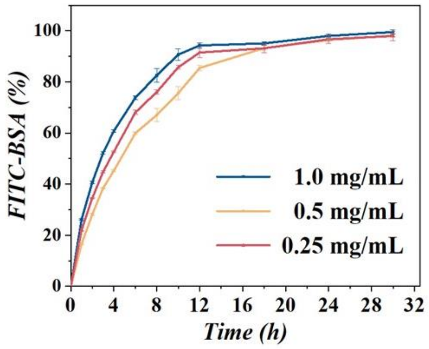


**Figure S8.** Drug release of GelMA/FITC-BSA with different concentrations of collagenase-II (1.0 mg $mL^{-1}$, 0.5 mg $mL^{-1}$, and 0.25 mg $mL^{-1}$).

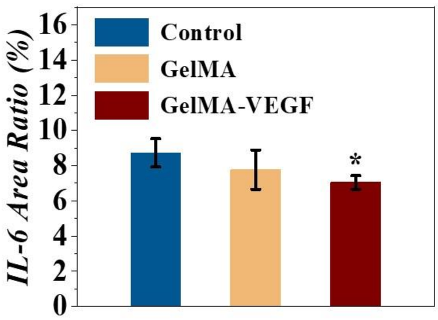


**Figure S9.** Quantitative analysis of the IL-6 on day 9, * $p < 0.05$ versus control group.
